# Effects of management of infection source of echinococcosis in Linzhi, Tibet Autonomous Region of China

**DOI:** 10.1186/s40249-021-00805-8

**Published:** 2021-03-06

**Authors:** Ying Wang, Bing-Cheng Ma, Li-Ying Wang, Gongsang Quzhen, Hua-Sheng Pang

**Affiliations:** 1 National Institute of Parasitic Diseases, Chinese Center for Disease Control and Prevention; Chinese Center for Tropical Diseases Research; WHO Collaborating Center for Tropical Diseases; National Center for International Research on Tropical Diseases, MOST; Key Laboratory of Parasite and Vector Biology, MOH, Shanghai, 200025 China; 2 Linzhi Center for Disease Control and Prevention, Linzhi, 850000 China; 3 Tibet Center for Disease Control and Prevention, Key Laboratory of Echinococcosis Prevention and Control, National Health Commission, Lhasa, 850000 China; 4 Doctorate School of Chemical and Biological Sciences for Health (CBS2), University of Montpellier, Montpellier, 34395 France

**Keywords:** Echinococcosis, Dog management, Dog infection, Linzhi, China

## Abstract

**Background:**

Echinococcosis is highly endemic in western and northern China. Tibet Autonomous Region (TAR) is the most serious prevalent area. Linzhi is located in southeastern part of TAR. Dogs are the primary infection source for the transmission of echinococcosis to humans. A control and prevention campaign based on dog management has been implemented in the past three years. This study aims to evaluate the effects of dog management on the infection rate of dogs.

**Methods:**

Data of dog population, registration and de-worming of seven counties/district in Linzhi between 2017 and 2019 were obtained from the annual prevention and control report. Domestic dog fecal samples were collected from each endemic town of seven counties/district in Linzhi in 2019 to determine the infection of domestic dogs using coproantigen enzyme-linked immunosorbent assay (ELISA). Data analysis was processed using SPSS statistics to compare dog infection rate between 2016 and 2019 by chi-square test, and maps were mapped using ArcGIS.

**Results:**

In Linzhi, domestic dog population has decreased from 17 407 in 2017 to 12 663 in 2019, while the registration rate has increased from 75.9% in 2017 to 98.6% in 2019. Similarly, stray dog population has decreased from 14 336 in 2017 to 11 837 in 2019, while sheltered rate has increased from 84.6% in 2017 to 96.6% in 2019. Dog de-worming frequency has increased from 4 times per annum in 2017 to 12 times in 2019, indicating that approximately every dog was dewormed monthly. A total of 2715 dog fecal samples were collected for coproantigen ELISA assay. The dog infection rate was 2.8% (77/2715) in 2019, which was significantly lower than 7.3% (45/618) in 2016 (*P* < 0.05).

**Conclusions:**

Increased dog registration, decreased dog population, and increased dog de-worming frequency contributed to significantly decrease the dog infection rate in Linzhi. Control and prevention campaign based on dog management could significantly decrease dog infection with *Echinococcus* spp. in echinococcosis endemic areas.
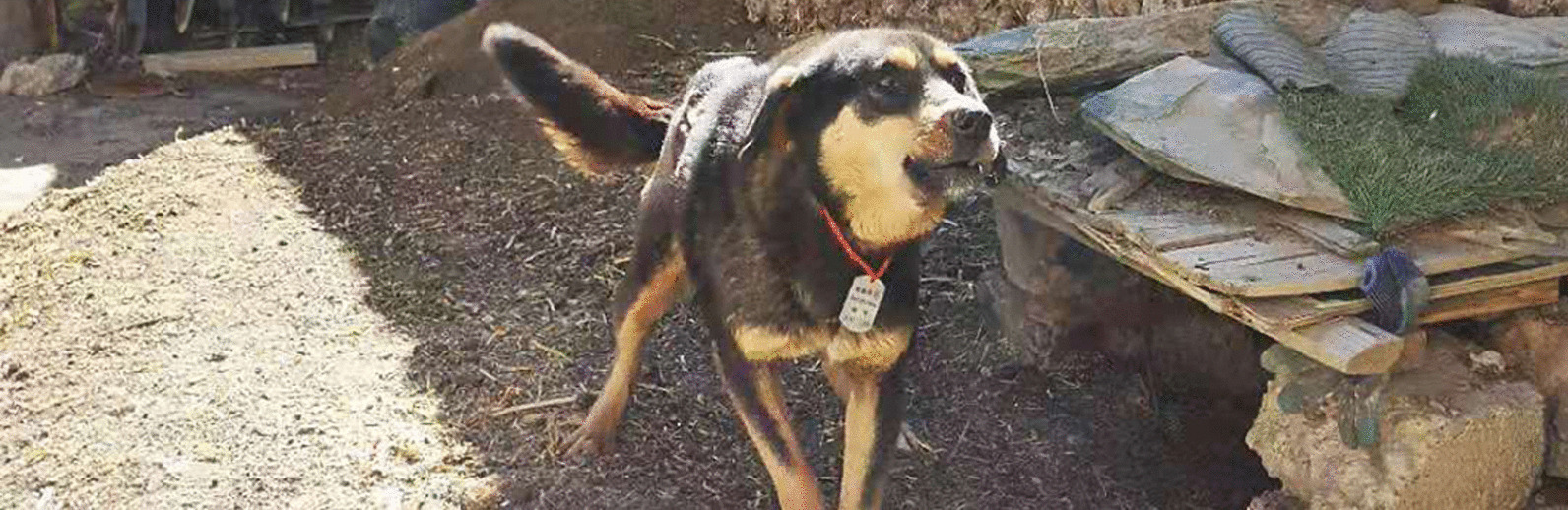

## Background

Echinococcosis is a severe zoonotic disease caused by the larval stage of the genus *Echinococcus*. Cystic echinococcosis (CE) caused by the metacestode of *Echinococcus granulosus* and alveolar echinococcosis (AE) caused by the metacestode of *Echinococcus multiocularis* are two forms of echinococcosis. Notably, CE is globally distributed while AE is majorly confined to the northern hemisphere [1, 2]. *Echinococcus* spp. has a complex life cycle that involves two hosts. The primary definitive hosts are dogs, which harbor adult worms in their small intestines. Humans and herbivores, specifically sheep, are the intermediate hosts of this parasite. The intermediate hosts become infected after ingesting the eggs released in the feces of definitive hosts. As the definitive hosts, dogs are pivotal in the transmission of echinococcosis [1].

Echinococcosis was found to be highly endemic in the pasture areas of northwestern China where the estimated prevalence of echinococcosis in humans was 0.3% (estimated prevalence was based on case detection rate in sample population), in livestock was 4.7%, and dog infection rate was 4.3% [3, 4]. Echinococcosis, threatening more than 50 million people in China, is among the key parasitic disease and a national control and prevention campaign was launched by Chinese central government since 2007 [5, 6]. Control and prevention of echinococcosis relies on de-worming of dogs with praziquantel, safe slaughtering conditions of livestock, and free screening and treatment of patients.

The Tibet Autonomous Region (TAR) of China is located in the Qinghai-Tibet Plateau, which has been reported as one of the most serious endemic regions across the globe [3, 4, 7]. Despite the control and prevention campaign bring launched over years, the prevalence of echinococcosis remains at a high level. Based on a 2016 epidemiological survey of echinococcosis in TAR, the estimated prevalence of echinococcosis in humans was 1.7%, in livestock was 11.8%, and dog infection rate was 7.3% [7]. As such, the government of TAR has strengthened control and prevention campaign of echinococcosis with increased financial support and additional technical guidance since 2017. Because of the ubiquity of dogs in TAR, increased attention has been channeled to dog management. Various measures have been implemented to strengthen the management of infection sources of echinococcosis, including dog registration, reduction of dog population, and the most importantly, monthly dog de-worming.

In order to evaluate the effects of dog management in the past three years in TAR, we collected data on dog registration, dog population, and dog de-worming from Linzhi, one of the seven prefectures in TAR. The feces of dogs from Linzhi were collected and the coproantigen enzyme-linked immunosorbent assay (ELISA) method was used to determine dog infection with *Echinococcus* spp., and the differences in dog infection between 2016 and 2019 were analyzed. The results of this work might boost the understanding of the efficacy of control and prevention campaigns in Linzhi and help other echinococcosis endemic areas.

## Methods

### Study area

Linzhi is located in the southeastern part of TAR between latitudes 26°52′–30°40′ N, and between longitudes 92°09′–98°47′ E. It is in the middle and lower reaches of the YarlungZangbo River with an average altitude of 3100 m, a total area of approximately 11.7 km^2^, and an overall population of 231 000. Linzhi has one administrative district and six counties, which are Bayi District, Gongbujiangda, Milin, Motou, Bomi, Chayu, and Lang County. Among these seven counties/district, Bayi, Gongbujiangda, Bomi, and Chayu were reported as co-endemic areas of both CE and AE, while the other three counties were reported as CE endemic areas [4, 7].

### Data collection

Data on dog population, registration and de-worming from 2017 to 2019 were obtained from the annual prevention and control report of Linzhi Center for Disease Control and Prevention, TAR.

### Management measures of dogs

#### Dog registration

For the convenience of management, a file has been created for each domestic dog, including the owner’s name, gender, age, and the date of each de-worming.

#### Dog de-worming

Monthly de-worming for domestic dog with praziquantel and each de-worming date were recorded on dog registration files. Dogs weighing less than 5 kg were given 50 mg each time; dogs weighing 5–15 kg were given 200 mg each time; and dogs weighing more than 15 kg were given 400 mg each time. Dog feces were buried in depth or burned after de-worming.

#### Reduction of dog population

Various measures have been taken to control dog population, including building shelters to contain stray dogs as many as possible, restricting domestic dogs to two individuals per household, leashing domestic dogs, etc. Data on domestic and stray dog population were collected by local veterinarians.

### Dog infection assay

One village was randomly selected from each endemic town of seven counties/district of Linzhi, and 20 households with dogs were randomly selected from each selected village based on the registration files of dogs. Only one dog fecal sample was collected from each selected household. To inactive any potential *Echinococcus* eggs, the collected fecal samples were frozen at − 80℃ for at least 72 h. All the dog fecal samples were tested for *Echinococcus* coproantigens using sandwich ELISA (Dog *Echinococcus* coproantigens ELISA kit, Combined, Shenzhen, China).

### Statistical analysis

Data analysis was performed using SPSS Statistics version 21.0 (IBM, New York, USA), geographic information maps were mapped using ArcGIS version 10.1 (ESRI, Redlands, USA). Dog infection rates in 2016 and 2019 were compared using chi-square test, and the level of statistical significance was set at *P* < 0.05.

## Results

### Increased dog registration rate

As in Table 1, registration rate of domestic dog was increased respectively each year from 75.9% (13 216/17 407) in 2017 to 98.6% (12 491/12 663) in 2019. Almost all the domestic dogs in each county/district have been registered for management. Domestic dog population has decreased from 17 407 in 2017 to 12 663 in 2019. The results showed that the dog management is strengthened gradually.

**Table 1:** Registration of domestic dogs in Linzhi in 2017–2019

County/district	2017			2018			2019		
	Number of domestic dog	Number of registered dog	Registration rate (%)	Number of domestic dog	Number of registered dog	Registration rate (%)	Number of domestic dog	Number of registered dog	Registration rate (%)
Bayi	3759	2722	72.4	2820	2679	95.0	2669	2643	99.0
GongbuJiangda	3759	3759	100.0	3310	3310	100.0	2305	2259	98.0
Milin	3287	1446	44.0	2651	2545	96.0	2168	2147	99.0
Motuo	1193	453	38.0	1054	1011	95.9	401	397	99.0
Bomi	2863	2863	100.0	2771	2660	96.0	2661	2608	98.0
Chayu	1912	1339	70.0	2126	1781	83.8	2048	2028	99.0
Lang	634	634	100.0	1780	1780	100.0	411	409	99.5
Total	17 407	13 216	75.9	16 512	15 766	95.5	12 663	12 491	98.6

### Increased dog de-worming frequency

In 2017, annual de-worming frequency was four times per annum, and the number increased to 11 in 2018 and 12 in 2019, which indicated that almost all domestic dogs had been de-wormed each month in 2019 (Table 2).

**Table 2:** De-worming frequency of domestic dogs in Linzhi in 2017–2019

County/district	2017			2018			2019		
	Number of domestic dog	Deworming doses (times)	Annual deworming frequency	Number of domestic dog	Deworming doses (times)	Annual deworming frequency	Number of domestic dog	Deworming doses (times)	Annual deworming frequency
Bayi	3759	14 460	4	2820	32 698	12	2669	29 853	11
GongbuJiangda	3759	13 514	4	3310	37 296	11	2305	26 901	12
Milin	3287	14 372	4	2651	30 544	12	2168	25 740	12
Motuo	1193	4162	3	1054	11 389	11	401	4684	12
Bomi	2863	11 452	4	2771	30 356	11	2661	31 474	12
Chayu	1912	7633	4	2126	23 806	11	2048	24 171	12
Lang	634	1759	3	1780	21 286	12	411	4721	11
Total	17 407	67 352	4	16 512	187 375	11	12 663	147 544	12

### Decreased stray dog population

So far, three shelters have been established for stray dogs in Linzhi, and stray dog population has gradually decreased from 14 336 in 2017 to 11 837 in 2019, while sheltered rate of stray dog was increased from 84.6% in 2017 to 96.6% in 2019. The results indicated that the vast majority of stray dogs have been sheltered by 2019 (Table 3).

**Table 3:** Stray dog population in Linzhi in 2017–2019

	2017			2018			2019		
County/district	Number of stray dogs	Number of sheltered dogs	Sheltered rate (%)	Number of stray dogs	Number of sheltered dogs	Sheltered rate (%)	Number of stray dogs	Number of sheltered dogs	Sheltered rate (%)
Bayi	3016	2684	89.0	2684	2518	93.8	2280	2166	95.0
GongbuJiangda	2914	2475	84.9	2486	2368	95.3	1751	1663	95.0
Milin	2064	1785	86.5	1954	1759	90.0	1780	1719	96.6
Motuo	1795	1468	81.8	1616	1437	88.9	1567	1536	98.0
Bomi	2715	2468	90.9	2684	2538	94.6	2986	2926	98.0
Chayu	986	684	69.4	917	846	92.3	855	825	96.5
Lang	846	568	67.1	726	597	82.2	618	603	97.6
Total	14 336	12 132	84.6	13 067	12 063	92.3	11 837	11 438	96.6

### Dog infection status

In 2019, a total of 2715 fecal samples were collected from domestic dogs in all seven counties/district in Linzhi, TAR. The dog infection rate in Linzhi was 2.8% (77/2715) in 2019, significantly lower than that in 2016, which was 7.3% (45/618) (*P* < 0.05). At the county level, the highest dog infection rate was 3.8% (40/1058) in Bayi district, followed by 3.4% (26/774) in Lang county, and the lowest dog infection rate was 0% (0/200) in Chayu county in 2019. There was a significant difference in dog infection between 2016 and 2019 in two county/district (Bayi district and Motuo county) (*P* < 0.05), and the other five counties showed no significant difference (*P* > 0.05) (Table 4).

**Table 4:** Dog infection in Linzhi between 2016 and 2019

County/district	2016				2019				*P-*value
	Total number of dogs	Number of dog fecal samples	Number of positive samples	Infection rate (%)	Total number of dogs	Number of dog fecal samples	Number of positive samples	Infection rate (%)	
Bayi	6775	117	27	23.1	2783	1058	40	3.8	< 0.05
GongbuJiangda	6673	81	4	4.9	2393	166	2	1.2	> 0.05
Milin	5351	80	1	1.3	2229	192	5	2.6	> 0.05
Motuo	2988	80	6	7.5	432	120	2	1.7	< 0.05
Bomi	5578	80	1	1.3	2721	205	2	1.0	> 0.05
Chayu	2898	100	3	3.0	2078	200	0	0.0	> 0.05
Lang	1480	80	3	3.8	426	774	26	3.4	> 0.05
Total	31 743	618	45	7.3	13 062	2715	77	2.8	< 0.05

### Dog infection distribution changes

**Fig. 1:**
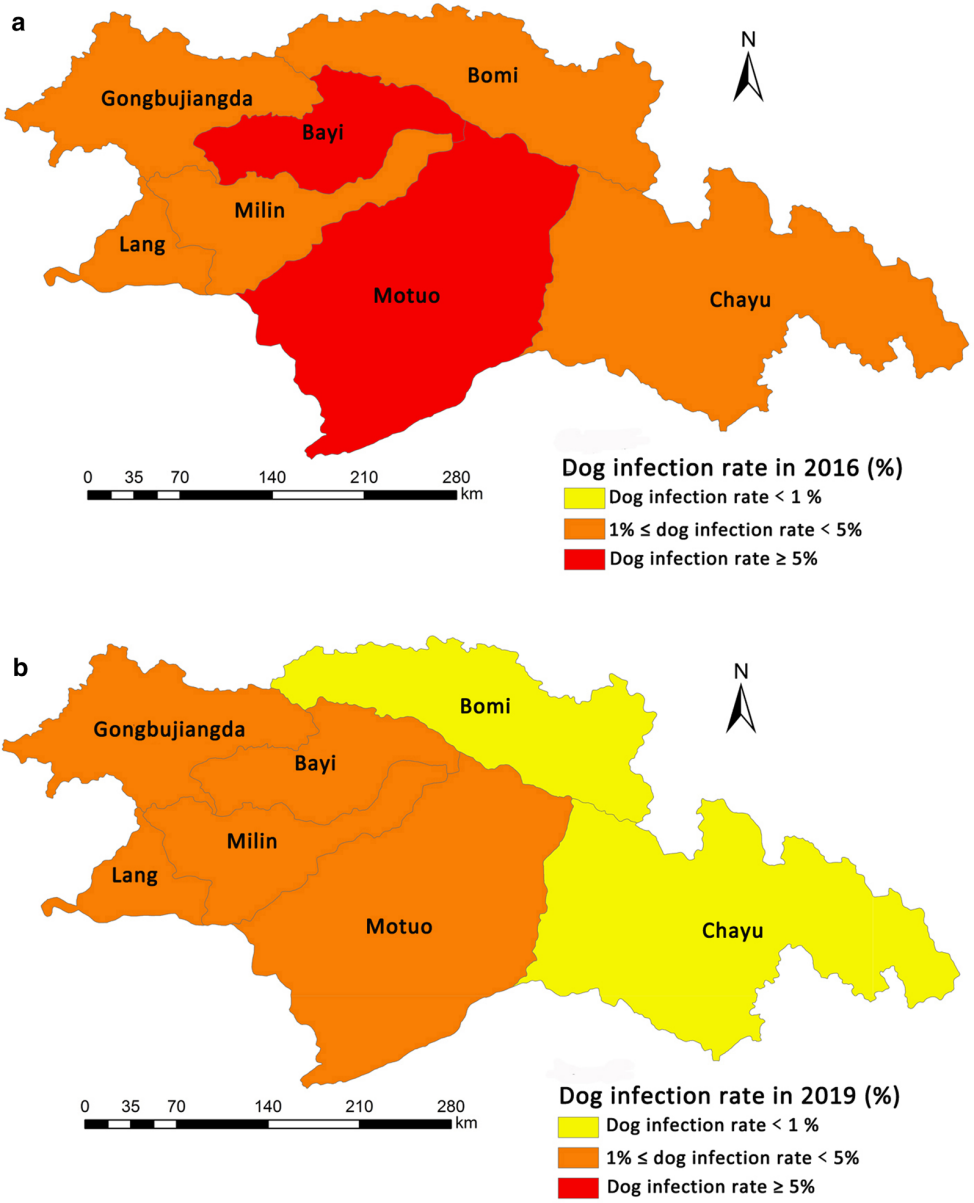
Dog infection distribution by county in Linzhi in 2016 (**a**) and 2019 (**b**)

According to national plan for echinococcosis and other key parasitic diseases prevention (2016–2020) [6], all echinococcosis endemic counties were categorized as three classes according to their prevalence in humans and dog infection rate. Class I (prevalence in humans ≥ 1% or dog infection rate ≥ 5%) is the most serious level. The epidemiological survey of echinococcosis in TAR in 2016 reported that the prevalence in humans in Linzhi was 1.6%, and the infection rate of dogs was 7.3% (45/618) [7]. As shown in Fig. 1, among seven counties/district of Linzhi, there were two counties were categorized as class I (Red in Fig. 1a) and the other five counties were categorized as class II (0.1% ≤ prevalence in humans < 1% or 1% ≤ dog infection rate < 5%) (Orange in Fig. 1a) in 2016. After three years with effort on control and prevention, two counties in Linzhi were down-categorized as class II, a total of five counties were categorized as class II (Orange in Fig. 1b), and the other two counties were down-categorized as class III (0 < prevalence in humans < 0.1% or 0 < dog infection rate < 1%) (Yellow in Fig. 1b) in 2019. The dog infection level of Linzhi in 2019 was significantly lower than that in 2016. This change illustrated that dog management can significantly decrease dog infection with *Echinococcus* spp.

## Discussion

The echinococcosis has been listed in 17 neglected tropical diseases by the World Health Organization (WHO) [8]. China, specifically the Qinghai-Tibet Plateau region, has the highest prevalence of echinococcosis across the globe [3, 4, 7]. Notably, the diseases are difficult to treat since it requires complex surgical procedures and long-term high dose anti-helminthic treatment, consequently, causing a heavy public health burden [9]. Additionally, the unique social-cultural background and lifestyle in Tibetan communities further complicates the control and prevention of the disease. Echinococcosis has been considered as a serious threat to public health in China, particularly in TAR.

Dog is the primary definitive host for *E. granulosus* and a major host for *E. multilocularis* if infected by ingesting small mammalian with metacestodes infection [9–12]. Due to their close relationship with humans, dogs are considered as the main risk to human echinococcosis [13–15]. Humans become infected via ingesting *Echinococcus* eggs released in the feces of dogs. Dog is an important part of most Tibetan pastoral families and communities, which resulted in large numbers of dogs around, including domestic and stray dogs. Therefore, how to reduce the dog population in the community and decrease dog infection are essential for echinococcosis control and prevention.

Domestic dogs in the Qinghai-Tibet Plateau, particularly those of herdsmen, participate in a wide range of activities and freely roam on the grassland. The feces of infected domestic dogs are scattered around the living areas of herdsmen, which can easily infect dog owners. Thus management measures for domestic dogs include leashing domestic dogs to minimize their roaming range, hence reducing their chance of infection, as well as restricting domestic dogs to two individuals per household to control their population and reduce the risk of transmission. Among those measures, de-worming dogs monthly is the most important one. All domestic dogs should be registered, and wear tags to distinguishing them, and each de-worming date recorded on the file to ensure maximum monthly de-worming. After de-worming, the dog feces should be deeply buried or burned to cut off the transmission route. By 2019, the registration rate of domestic dogs in Linzhi was 98.6%, the number of domestic dogs had gradually decreased (Table 1), and the annual de-worming frequency had reached to 12 times (Table 2). The infection rate of dogs in Linzhi in 2019 was significantly lower than that in 2016. All the above data showed that these management measures were effective.

Dogs that are not leashed, without tags and not registered are considered as stray dogs. Stray dogs usually have a larger range of activities compare to domestic dogs and are more likely to be infected by ingesting internal organs of infected livestock and small mammalian. Furthermore, the feces of infected stray dogs are scattered in the surrounding areas, which made stray dogs more important for the transmission. The most important management measure for stray dogs is to contain them as much as possible, and de-worming of sheltered stray dogs is essential too. The feces of sheltered dog are deeply buried or burned after de-worming to reduce environmental contaminant. Table 3 showed that by 2019, the vast majority (96.6%) of stray dogs were contained in shelters and dewormed, there were only a few stray dogs in surrounding areas. Decreased number of stray dogs greatly reduced the transmission risk of echicococcosis. Therefore, the infection rate of stray dogs were undetected in this study.

Meanwhile, we found that despite the dog infection rate significantly decreasing in Linzhi, it remained at a relatively high level. In some counties, dog registration, reduction of dog population and dog de-worming have been effectively conducted, and the vast majority of dogs had been registered and de-wormed monthly by 2019. Nonetheless, the dog infection still remained high, which seems to be a contradiction to the results. The coproantigen ELISA method has been used to determine dog infection and may have cross reaction with other cestode infection. False positive caused by this [2, 16] may be one of the reasons why infection rate remained relatively high. Moreover, there might have been a few hurdles during the implementation of dog de-worming since it was difficult to ensure that dogs effectively take praziquantel during the process, which may lead to ineffective de-worming.

Annually surveillance on dog population, de-worming frequency and dog infection rate in echinococcosis endemic areas can accurately evaluate the effects of management measures of infection source. In this work, the annual data on dog population and de-worming frequency were collected, but the dog infection rate was only in 2016 and 2019, so it was difficult to draw the trend of dog infection and prevalence of echinococcosis, it also suggests that more attention should be paid to annually surveillance and data management in future work, so as to more effectively evaluate the effect of various prevention and control measures and predict the trend of prevalence of echinococcosis in endemic areas.

## Conclusions

This work described the changes in dog registration, dog population and dog de-worming frequency in Linzhi, TAR between 2017 and 2019, and compared dog infection between 2016 and 2019. Our finding revealed that increased dog registration rate, decreased dog population and increased dog de-worming frequency contributed to significantly decreased dog infection rate in Linzhi, TAR. Control and prevention campaign based on dog management can significantly decreases dog infection with *Echinococcus* spp. in echinococcosis endemic areas.

## Data Availability

All relevant data can be found within this paper.

## Abbreviations

AE: Alveolar echinococcosis
CE: Cystic echinococcosis
ELISA: Enzyme-linked immunosorbent assay
TAR: Tibet Autonomous Region
WHO: World Health Organization
